# Prevalence of SARS-CoV-2 antibodies in the Palestinian population: A primary health center-based cross-sectional study

**DOI:** 10.1371/journal.pone.0258255

**Published:** 2021-10-08

**Authors:** Beesan Maraqa, Walid Basha, Rasha Khayyat, Abdul-Rahman Abdul-Hadi, Jurouh Jabareen, Kamal Al-Shakhra, Mai Al-Kaila, Zaher Nazzal

**Affiliations:** 1 Primary Health Directorate, Ministry of Health, Nablus, Palestine; 2 Department of Biomedical Sciences, Faculty of Medicine and Health Sciences, An-Najah National University, Nablus, Palestine; 3 Ministry of Health, Nablus, Palestine; 4 Department of Medicine, Faculty of Medicine and Health Sciences, An-Najah National University, Nablus, Palestine; Bangalore Baptist Hospital, INDIA

## Abstract

This study aimed to assess the prevalence of severe acute respiratory syndrome coronavirus-2 (SARS-CoV-2) total antibodies in the north, middle, and south regions of West Bank and the prevalence of SARS-CoV-2 specific antibodies (IgA, IgM, and IgG) in the Palestinian population. This was a cross-sectional study. The serological and epidemiological data of 1269 persons were assessed. Participants were selected randomly among primary health care center attendees in Palestine between November 1, 2020 and December 31, 2020. All serum samples were tested for total antibodies using an enzyme-linked immunosorbent assay (ELISA) test. IgM, IgG, and IgA-specific antibody titers were measured using ELISA. The overall prevalence (with 95% confidence intervals [CIs]) of SARS-CoV-2 total antibodies and specific antibodies were estimated. A multivariate regression model was used to assess the predictive factors for SARS-CoV-2-specific antibodies. The overall seroprevalence of SARS-CoV-2 antibodies was 24·0% (95% CI, 21·7%–26·5%). Seroprevalence was significantly higher among people living in south West Bank (adjusted Odds ratio [aOR], 2·22; 95% CI: 1·58–3·11), people who had COVID-19 symptoms (aOR, 3·92; 95% CI, 2·83–5·43), people with a COVID-19 contact history (aOR, 1·44; 95% CI, 1·03–2·03), patients with hypertension (aOR, 1·57; 95% CI, 1·06–2·33), and non-smokers (aOR, 0·47; 95% CI, 0·31–0·72). A total of 171 blood samples from SARS-CoV-2-positive patients were chosen at random for additional serological testing. Specific IgM, IgG, and IgA antibodies were positive in 14·0% (95% CI, 9·2%–20·2%), 88·3% (82·5%–92·7%), and 42·1% (34·6%–59·9%) of the samples, respectively. SARS-CoV-2 antibodies were common among PHC center attendees and were significantly associated to sex, smoking, and COVID-19 contact history. However, considering that almost three-quarters of this population remains susceptible, maintaining public health measures and encouraging access to immunization is critical in protecting this population.

## Introduction

Coronavirus disease (COVID-19) was first diagnosed in December 2019 in China. The World Health Organization (WHO) declared it a pandemic on March 11, 2020 [1, 2]. As of February 8, 2021, WHO reported 105,805,951 confirmed COVID-19 cases and 2,312,278 deaths [3].

Primary health care (PHC) centers are the first level of contact for individuals, families, and communities [4]. In Palestine, a network of over 424 PHC centers is distributed in the West Bank. The PHC centers address the major health problems in the community and offer preventive, curative, and rehabilitation services. According to the Palestinian Health Report of 2019, the overall number of visits to PHC physicians in the West Bank was 2,149,486, while the number of visits to PHC nurses was 2,208,190 [5].

By the end of December 2020, approximately 140,000 laboratory-confirmed severe acute respiratory syndrome coronavirus-2 (SARS-CoV-2) infection cases were registered in the West Bank of Palestine. However, these results are only a fraction of the total number of SARS-CoV-2 infections, given that not all infected persons are symptomatic [6, 7]. Asymptomatic patients may be infectious, and thus a likely source of COVID-19 transmission for a long period [8, 9].

In Palestine, only people with clinical symptoms or close contacts are tested; this gives a lower SARS-CoV-2 infection rate than the actual rate. To overcome these limitations and understand population-level infection data, serology testing is important [10]. It is an important addition to detection and diagnosis using polymerase chain reaction (PCR) [11]. In addition, anti-SARS-CoV-2 IgG/IgM/IgA detection in the general population can be a good indicator of exposure to SARS-CoV-2 [12–15].

Total serum antibody levels are associated with clinical disease severity; that is, higher levels are associated with more severe disease. This implies that it is important to assess patients’ immune status [16]. Research on immune responses to COVID-19 has been limited to serum antibodies, systemic cell-mediated immunity, and innate responses. Mucosal immunity, secretory IgA antibodies, and circulating IgA antibodies are implicated in COVID-19 [17]. IgA is the primary antibody involved in mucosal immunity, and it is the most effective immunoglobulin against infectious pathogens in the respiratory and digestive systems [18]. Multiple studies have shown that the humoral immune response worsens COVID-19 symptoms, inflammation, and mortality, most likely through antibody-dependent enhancement [19].

WHO recommends the use of seroprevalence of SARS-CoV-2 antibodies to assess the rate of disease transmission in the general population and its subgroups [4]. Since April 2020, seroprevalence studies have been conducted in several countries [12, 14, 15, 20–22]. Globally, estimates of SARS-CoV-2-specific antibodies have shown variability in the general population [15]. The different time frames also play a role in seroprevalence. In the United States of America, seroprevalence ranges from less than 1% to 23% over six months [21]. Additionally, seroprevalence varies with sex, age group, and geographical location [23, 24].

The level of SARS-CoV-2 specific antibodies is crucial in the assessment of the pandemic potential of COVID-19, success of public health interventions, and planning for the implementation of additional preventive and control measures [25]. However, the overall seroprevalence of SARS-CoV-2 and risk factors in Palestine are unknown. The primary objectives of this study were to determine (1) the prevalence of SARS-CoV-2 total antibodies in the north, middle, and south regions of the West Bank and (2) the prevalence of SARS-CoV-2-specific antibodies (IgA, IgM, and IgG) among Palestinians. The secondary objectives were to assess (1) the correlation of the level of total antibodies with the population’s background and clinical characteristics and (2) the correlation of IgA and IgG levels with participants’ signs and symptoms.

## Materials and methods

### Study design and population

This was a comparative cross-sectional study. Serological testing for anti-SARS-CoV-2 antibodies were used to assess the prevalence of SARS-CoV-2 immunity. A systematic random sampling technique was used. Primary health care center attendees for more than one month were considered as the entire study population. The anticipated number of attendees per district per day was calculated. Further, the required sample per day was calculated to obtain the (x) factor for systematic sampling intervals. The study was explained to every xth person of our target population, inviting them to participate in the research. The study was conducted between November 1, 2020 and December 31, 2020.

As an occupied country, Palestine comprises the West Bank, including East Jerusalem and the Gaza Strip. Since the authorization for this study was limited to the West Bank, seroprevalence was calculated only for the West Bank. PHC centers are located in all governorates, all of which are public centers under the Palestinian Ministry of Health administration. They are typically the first point of contact for most residents in those areas and health facilities, both for curative and preventive services. People who visited the PHC centers in all West Bank governorates (Jenin, Tubas, Tulkarm, Qalqilia, Nablus, Salfit, Ramallah, Jericho, Jeruzalem, Bethlehem, and Hebron) for different reasons, excluding children and adolescents (age <18 years) comprised the study population.

The sample size needed to achieve the research objectives and adequate statistical power was calculated using the following equation: *n* = [DEFF*Np(1-p)]/ [(d^2^/Z^2^_1-α/2_*(N-1)+p*(1-p)]. The Palestinian population as recorded during the 2017 census was approximately five million in the West Bank and Gaza, with approximately three million in the West Bank [26]. To compare different geographical areas in Palestine, the West Bank was divided into three regions: the north, middle, and south; the sample size was calculated for each region. Using a population size *(N)* of one million, % frequency of the outcome factor in the population *(p)* = 50% ±5, confidence limits as 100% *(d)* = 5%, design effect *(DEFF)* = 1, and 95% confidence interval, the calculated sample size *(n)* for each of the West Bank regions was 384. Therefore, the minimum acceptable total sample size was 1150. The sample size needed for each city was proportional to the population of each city, relative to the total population of all the cities included.

### Ethical considerations

All the procedures performed in this study complied with federal and institutional ethical standards, the 1964 Helsinki Declaration, and subsequent amendments or equivalent ethical standards. The study was approved by the Institutional Review Board (IRB) committee of An-Najah National University (Reference #: Med 4/20/2) and the Palestinian Ministry of Health. People who visited the PHC centers were invited to participate in the study voluntarily, and those who agreed signed an informed consent form. The study background and objectives were explained in detail to the participants. The results of the participants’ blood investigations were delivered via the PHC directorates.

### Measures

The study participants were asked various questions about their demographic and clinical characteristics. The demographic characteristics included age, sex, residency, marital status, and occupation. The clinical characteristics included the presence of any comorbidity, COVID-19 symptoms, previous diagnosis of COVID-19, and COVID-19 contact history.

Approximately 5 ml of venous blood were collected from each study participant by a qualified laboratory technician at each PHC center. The blood was centrifuged for serum separation, and the serum was transported to the research laboratory of the Faculty of Medicine and Health Sciences at An-Najah National University (ANU). Samples were kept at -80°C in sterile microtubes until the enzyme-linked immunosorbent assay (ELISA) test was performed, according to the manufacturer’s instructions. All serum samples were anonymized and tested for total antibodies using ELISA (Elecsys^®^ Anti-SARS-CoV-2, Roche Diagnostics Ltd). According to the manufacturer’s recommendations, samples were considered positive above a cutoff index of 1 for total antibodies. A random sample of the total antibody-positive serum samples was selected to measure the titers of the specific antibodies: IgM, IgG, and IgA. ELISA was used for the qualitative detection of IgG, IgM, and IgA antibodies to SARS-CoV-2 using EUROIMMUN Anti-SARS-CoV-2 ELISA, Anti-SARS-CoV-2 NCP ELISA, and Anti-SARS-CoV-2 ELISA, respectively. According to the manufacturer’s recommendations, samples were considered positive above Nova Tec units (NTU) 1·1 for IgM, IgG, and IgA antibodies. The reported clinical sensitivities/specificities by the manufacturer was 100%/99·6%, 57·1%/97·2%, and 100%/93·8% for IgG, IgM, and IgA, respectively.

### Statistical analysis

All statistical analyses were performed using IBM SPSS Statistics for Windows, Version 20·0 (IBM Corp., Armonk, NY: IBM Corp). Continuous variables are expressed as mean ± standard deviation (SD). Categorical variables are described as counts and percentages. We estimated the overall prevalence of SARS-CoV-2 total antibodies and IgM, IgG, and IgA-specific antibodies with 95% confidence intervals. The chi-square test was used to compare categorical variables of SARS-CoV-2 specific antibody-positive and -negative groups and to establish associations. The likelihood-ratio chi-square test was used to assess the relationship between SARS-CoV-2 serology and COVID-19 symptoms. A stepwise multivariate regression model was used to evaluate the factors predicting SARS-CoV-2 specific antibody seropositivity. Statistical significance was defined as a two-sided P value of <0·05.

## Results

### Background characteristics

Between November and December 2020, 1360 blood samples were collected from people attending PHC centers in the West Bank. However, 91 people were excluded from the study for a variety of reasons, including an incomplete survey, being under the age of 18, and improperly collected blood samples, resulting in a total sample size of 1269 people. The age distribution of the study population was consistent with that of the Palestinian population. Of the 1269 participants, 520 (40%) were men. Most of them (75·7%) were married and non-smokers (72%) (Table 1).

**Table 1:** Background characteristics according to SARS-CoV-2 seroprevalence (n = 1269).

Variable	COVID-19 Antibodies			
	Negative (n = 964)	Positive (n = 305)	Total (n = 1269)	P Value
***Age (years)***				
≤30	318 (82.4%)	68 (17.6%)	386 (30·4%)	·001
≥31 and <60	494 (72·2%)	190 (27·8%)	684 (53.9%)	
≥60	152 (76.4%)	47 (23.6.2%)	199 (15.7%)	
***Sex***				
Male	421 (83·0%)	86 (17·0%)	507 (40·0%)	<·001
Female	543 (71·3%)	219 (28·7%)	762 (60·0%)	
***Residence***				
North	456 (83·1%)	93 (16·9%)	549 (43·3%)	<·001
Middle	224 (77·5%)	65 (22.5%)	289 (22·7%)	
South	284 (65.9%)	147 (34·1%)	431 (34·0%)	
***Residency setting***				
Urban	389 (74.1%)	136 (25.9%)	525 (41·9%)	·185
Rural	563 (77·3%)	165 (22·7%)	728 (58·1%)	
***Marital status*** *				
Single	230 (80.1%)	57 (19·9%)	287 (24·3%)	·059
Married	733 (74·7%)	248 (25·3%)	981 (75·7%)	
***Smoking***				
Non-smoker	653 (72·2%)	252 (27.8%)	905 (71·6%)	<·001
Current smoker	278 (85·8%)	46 (14·2%)	324 (25·6%)	
Ex-smoker	31 (68·1%)	5 (13·9%)	36 (2·8%)	
***Occupation***				
Health care setting	99 (70·2%)	42 (29·8%)	141 (11·6%)	·006
Government	90 (79.8%)	39 (30·2%)	129 (10·6%)	
Education sector	74 (75.5%)	24 (24.5%)	98 (8.1%)	
Active worker	237 (83·5%)	47 (16·5%)	284 (23·4%)	
Unemployed	422 (74.8%)	142 (24·5%)	564 (46·4%)	
***Chronic diseases***				
Yes	341 (71·5%)	136 (28·5%)	477 (37·8%)	·004
No	616 (78·6%)	168 (21·4%)	784 (62·2%)	
***Diabetes mellitus***	190 (70·6%)	79 (29·4%)	270 (20·8%)	·021
***Hypertension***	199 (69·1%)	89 (30·9%)	269 (21.2%)	·002
***Cardiovascular disease***	071 (72.4%)	27 (27·6%)	98 (7·7%)	·396
***Respiratory diseases***	035 (67·3%)	17 (32·7%)	52 (4·1%)	·136
***Previous COVID-19 diagnosis*** *				
Yes	27 (22.9%)	91 (77.1%)	118 (9·6%)	<·001
No	904 (81.0%)	212 (19·0%)	1116 (90·4%)	
***COVID-19 contact history*** *				
Yes	242 (64·4%)	134 (35.6%)	376 (29·8%)	<·001
No	714 (80·7%)	171 (19·3%)	885 (70·2%)	
***COVID-19 symptoms*** *				
Yes	190 (54.8%)	157 (45·2%)	347 (27·8%)	
No	756 (83·7%)	147 (16·3%)	903 (72·2%)	<·001

*Not adding up to 1269 due to missing data; COVID-19 = coronavirus disease; SARS-CoV-2 = severe acute respiratory syndrome coronavirus-2.

### SARS-CoV-2 total antibodies and associated factors

**Fig 1:**
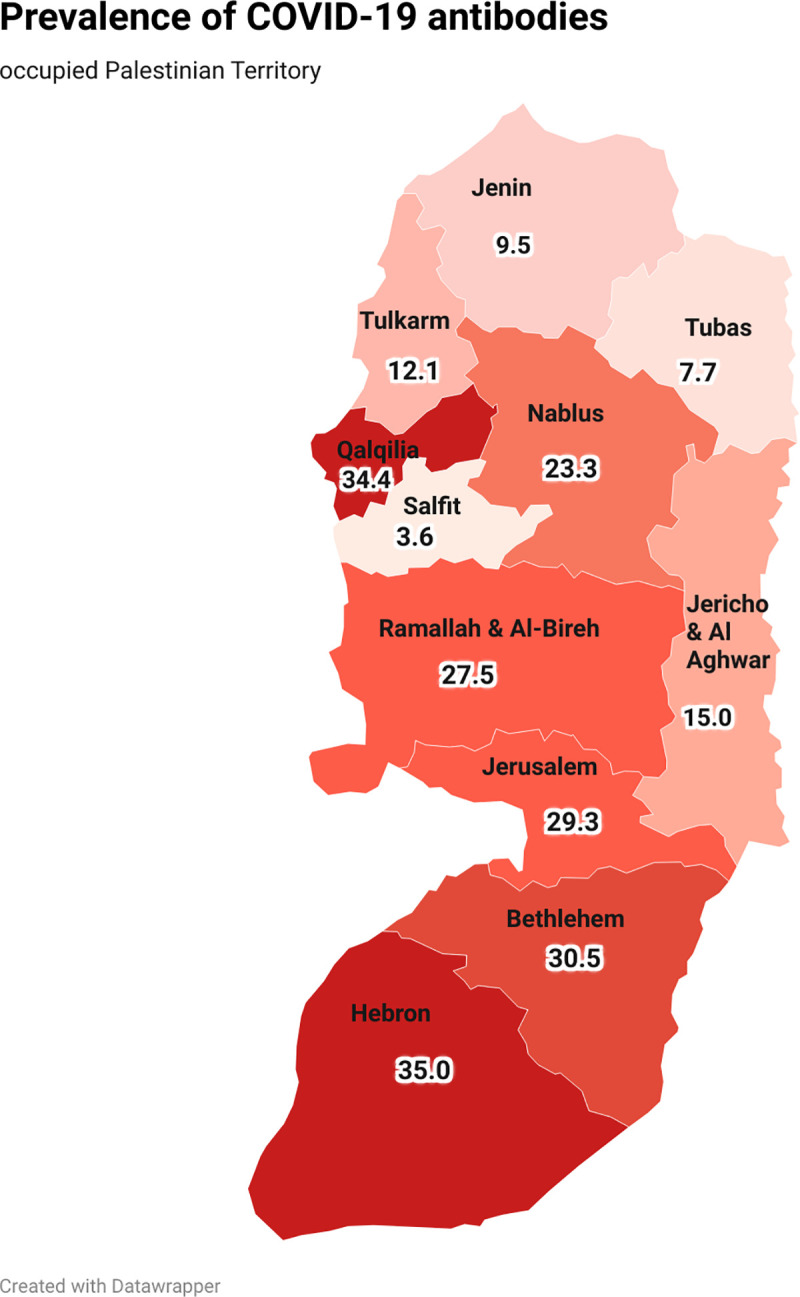
Serologically reported cases of severe acute respiratory syndrome coronavirus-2 (SARS-CoV-2) in the general population of Palestine between November 1, 2020 and December 30, 2020. “Reprinted from [27] under a CC BY license, with permission from [datawrapper], original copyright [2021].

A total of 305/1269 patients tested positive for SARS-CoV-2 (24·0% [95% CI 21·7%–26·5%]). As shown in Fig 1, the prevalence of antibodies was higher in the areas on the Israeli border, particularly in Hebron and Qalqelia, where the prevalence of total SARS-CoV-2 antibodies was more than 30% of the total population. The prevalence was lower in the north regions, notably Jenin and Tubas (9·5% and 7·7%, respectively).

SARS-CoV-2 seroprevalence varied with multiple variables. The highest level of positive antibody response was recorded in the middle-age group (p ·001). This was same among the females. Likewise, seroprevalence positivity was high among those who lived in the south region of the West Bank. There was a significant difference in seroprevalence between non-smokers and smokers. In addition, those working in healthcare settings and multi-interaction settings (citizens with the government and education) had a high level of SARS CoV-2 antibodies (Table 1).

The presence of chronic illnesses like hypertension and diabetes influences the prevalence of SARS-CoV antibodies. Patients with chronic illnesses had a higher level of antibodies. Patients diagnosed with COVID-19 who were PCR-positive had a higher level of antibodies. The longest period of antibody positivity following diagnosis was six months (May 1, 2020 to November 1, 2020). COVID-19 contact and symptoms were significantly associated with presence of anti-SARS-CoV-2 antibodies (Table 1). Of those who were tested positive for SARS-Cov-2 antibodies, 70·7% were not previously diagnosed with COVID-19. About half of those who were tested positive for SARS-Cov-2 antibodies had COVID-19 symptoms, and 43·6% had a COVID-19 contact history.

### Multivariate analysis of factors associated with SARS-CoV-2 antibodies

People living in the south (P, <·001; aOR, 2·22; 95% CI, 1·58–3·11] or middle (P, ·021; aOR, 1.58; 95% CI, 1·11–2·34] of the West Bank were more likely to have positive antibodies than those living in the north. Patients who had COVID-19 symptoms were 3.9 times more likely to be seropositive for SARS-CoV-2 (P, <·001; aOR, 3·92; 95% CI, 2·83–5·43), and patients with a COVID-19 contact history were 1·4 times more likely to show an antibody response (P, ·038; aOR, 1·48; 95% CI, 1·03–2·03]. There was a significantly higher positive antibody response among patients with hypertension (P, ·026; aOR, 1·57; 95% CI, 1·06–2·33). Current smokers were less likely to have SARS-CoV-2 antibodies than non-smokers (P, <0·001; aOR, 0·472; 95% CI, 0·31–0·72) (Table 2).

**Table 2:** Multivariate model of factors independently associated with SARS-CoV-2 seroprevalence.

	P value*	*Adjusted OR (95% CI)*
**Age (years)**		
≤30^†^		
31–60	·055	1.42 (0·99–2·03)
≥60	·575	1·17 (0·67–2. 03)
**Sex**		
Male†		
Female	·138	1.35 (0·91–2.02)
***Occupation***		
Health care setting	·069	0·62 (0·37–1·04)
Governmental sector	·719	0·91 (0·53–1·56)
Education sector	·522	1·20 (0·68–2·13)
Active workers	·982	0·99 (0·61–1·163)
Unemployed^†^		1
***Residence***		
North^†^		
Middle	·021	1·58 (1·11–2·34)
South	<·001	2·22 (1·58–3·11)
***Smoking***		
Non-smoker^†^		
Current smoker	<·001	0·47 (0·31–0·72)
Ex-smoker	·070	0·34 (0·11–1·09)
***COVID-19 contact history***		
Yes^†^	·038	1·44 (1·03–2·03)
No		
***COVID-19 symptoms***		
Yes	<·001	3·92 (2·83–5·43)
No^†^		
***Diabetes mellitus***		
Yes	·580	1·12 (0·74–1.69)
No^†^		
***Hypertension***		
Yes	·026	1·57 (1·06–2·33)
No^†^		

**OR:** odds ratio, **CI:** confidence interval.

Of the assessed COVID-19 symptoms, only fever, loss of taste and smell were significantly correlated with seroprevalence of SARS-CoV-2-specific antibodies. Likewise, the presence of muscle aches in the last six months was not an independent factor for seropositivity (Table 3).

**Table 3:** SARS-CoV-2 serology and COVID-19 symptoms.

Symptom	Negative (n%)	Positive (n%)	Total (n%)	LR*	Adj. OR	Adj. P-Value
***Fever***						
Yes	81 (48·8%)	85 (51·2%)	166 (13·1%)	67·4	1·8	·017
No	883 (80·1%)	220 (19·9%)	1103 (86·9%)			
***Cough***						
Yes	98 (55·1%)	80 (44·9%)	178 (14·0%)	44·2	·82	·47
No	866 (79·4%)	225 (20·6%)	1091 (86·0%)			
***Sore throat***						
Yes	83 (57·6%)	61 (42·4%)	144 (11·3%)	26.7	·58	·074
No	881 (78·3%)	244 (21·7%)	1125 (88·7%)			
***Diarrhea***						
Yes	37 (43·5%)	48 (56·5%)	85 (6·7%)	44·3	4·6	·65
No	927 (78·3%)	257 (21·9%)	1184 (93·3%)			
***Dyspnea***						
Yes	57 (46·7%)	65 (53·3%)	122 (9·6%)	54·3	1·15	·64
No	907 (79·1%)	240 (20·9%)	1147 (90·4%)			
***Headache***						
Yes	123 (53·9%)	105 (46·1%)	228 (18·0%)	63·3	1·50	·140
No	841 (80·8%)	200 (19·2%)	1041 (82·0%)			
***Ageusia***						
Yes	20 (21·7%)	72 (78·3%)	92 (7·2%)	132	3·50	·002
No	944 (80·2%)	233 (19·8%)	1177 (92·8%)			
***Anosmia***						
Yes	15 (18·3%)	67 (81·7%)	82 (6·5%)	132	4·09	·001
No	949 (79·9%)	238 (20·1%)	1187 (93·5%)			
***Muscle ache***						
Yes	93 (48·4%)	99 (51·6%)	192 (15·1%)	82	1·68	·051
No	871 (80·9%)	206 (19·1%)	1077 (84·9%)			

* The likelihood ratio chi-square, **Adj. OR:** adjusted odds ratio.

**Fig 2:**
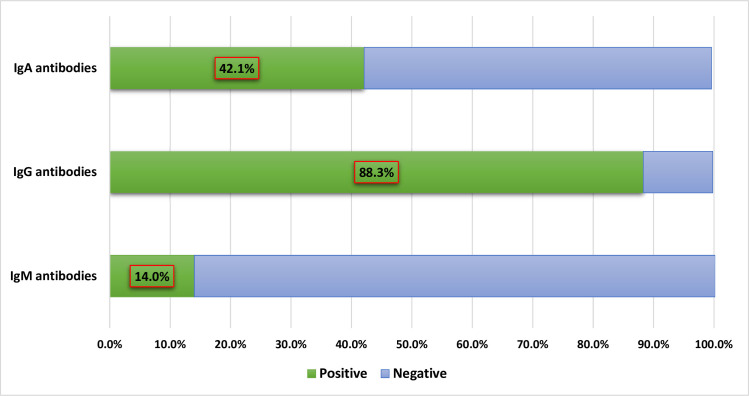
The prevalence of IgM, IgG, and IgA-specific antibodies in a random sample of SARS-CoV-2 samples (n = 171).

Of the SARS-CoV-2-positive blood samples, 171 were selected randomly for further serological testing. Twenty-four (14·0% [95% CI, 9·2%–20·2%]) were IgM-positive, 151 (88·3% [95% CI, 82·5%–92·7%]) were IgG-positive, and 72 (42·1% [95% CI, 34·6%–49·9%]) were IgA-positive (Fig 2). The period of detection of specific antibodies after diagnosis varied: 10 days–4 months for IgM, 17 days–6 months for IgG, and 10 days–6 months for IgA. It is worth noting that 7% of the samples were negative for either antibody.

The multivariate analysis showed that IgA development was significantly higher in men and older age groups (Table 4).

**Table 4:** Distribution of IgG and IgA specific antibodies according to background and clinical characteristics (n = 171).

Variable	COVID 19 Antibodies				
	IgG-Positive (n = 154)	P Value	IgA-Positive (n = 72)	P Value	Adjusted P value
***Age (years)***					
≤30^†^	25/33 (75·8%)	·036	5/33 (15·2%)	<·001	·005
31–60	105/116 (90·5%)		52/116 (44·8%)		
					·244
≥60	21/22 (95·5%)		15/22 (68·2%)		
***Sex***					
Male	39/42 (92·9%)	·260	24/42 (57·1%)	·022	·037
Female^†^	112/139 (86·8%)		48/129 (37·2%)		
***Smoking***					
Non-smoker	128/145 (88·3%)	·886	61/145 (42·6%)	·641	--
Current smoker	21/24 (83·9%)		9/24 (37·5%)		
***Chronic diseases***					
Yes	72/77 (93·5%)	·052	43/77 (55·8%)	·001	·161
No^†^	78/93 (83·9%)		28/93 (30·1%)		
***Previous COVID-19 diagnosis***					
Yes	51/56 (91·1%)	·454	24/56 (42·9%)	·903	--
No	99/114 (86·8%)		47/114 (41·9%)		
***COVID-19 contact history***					
Yes	72/79 (91·1%)	·295	30/79 (38·0%)	·216	---
No	79/92 (85·9%)		42/92 (45·7%)		
***COVID-19 symptoms***					
Yes	77/87 (88·5%)	·933	31/87 (35·6%)	·086	·607
No^†^	74/84 (88·1%)		41/84 (48·8%)		
***Fever***	41/46 (89·11%)	·877	19/46 (41·3%)	·845	
***Diarrhea***	25/26 (96·2%)	·185	12/26 (46·2%)	·685	
***Dyspnea***	33/35 (94·3%)	·230	17/35 (48·6%)	·418	
***Anosmia***	35/39 (89·7%)	·783	14/39 (35·9%)	·342	
***Ageusia***	36/40 (90·0%)	·736	15/40 (37·5%)	·464	
***Muscle ache***	50/56 (89·3%)	·824	18/56 (32·1%)	·056	

^†^Reference Group, **Adj. OR:** adjusted odds ratio.

## Discussion

Seroprevalence studies are commonly used to track the levels of immunity or presence of particular diseases within a population. This allows countries to prioritize and establish targeted strategies to reverse epidemics of non-communicable diseases [28, 29]. Total antibodies, which are reflective of group immunity, were found in 24.0% of the Palestinian population nine months after the pandemic. Given the previous SARS-CoV experience, this suggests that protection against coronavirus in Palestinians lasts for at least one year and may reach six years [30]. An increase in the knowledge on herd immunity may be ramification of population transmission. WHO recommended programmed vaccination as a first step to achieving vaccine-induced herd immunity [31]; however, this was not possible in low-income countries such as Palestine, in which the vaccines were not accessible at the time of the recommendation (March 2021).

Compared to previous studies performed in other countries, the prevalence of seropositive antibodies was higher. Moreover, the spatial distribution of immunity in this study was expected [21]. Since the study was designed to obtain a representative sample at the national level, geographic variations at the district level were observed. The findings were generally consistent with the national surveillance data at the time of data collection [32].

In Palestine, the spatial distribution of SARS-CoV-2 antibody prevalence has substantial causation, indicating the significance of preventive measures. The south West Bank is known for its strong social ties, which cannot be avoided. These include wedding and funeral traditions, such as large gatherings to congratulate celebrants and sharing of a meal with several people. Moreover, due to the political situation in Palestine, many young men are employed in Israel. This plays an essential role in the initial spread surge within the population [33].

Of the people with a positive antibody test, approximately two-third of them was not diagnosed with COVID-19 previously at some point during the outbreak. Asymptomatic individuals can serve as sources of SARS-CoV-2 infection [34]. Thus, the exposed population may have been larger than the reported population. Different serology studies have estimated the incidence of SARS-CoV-2 infection in asymptomatic people. In Brazil, 11·9% of asymptomatic individuals were IgM-positive, and 8·3% were IgG-positive [20]. Another study showed that the total number of COVID-19 cases was 6–24 times greater than the number of confirmed cases [21].

Smoking and COVID-19 has become a popular topic in this era. In our study, current smokers had significantly lower antibody levels than non-smokers. In a longitudinal study, smokers had lower antibody titers than non-smokers over time. This may have accounted for the disease duration [35]. A living evidence review showed that current smokers had a decreased risk of SARS‐CoV‐2 infection than never-smokers, while former smokers had a greater risk of hospitalization, increased disease incidence, and COVID‐19 mortality than never-smokers [36]. Studies have consistently shown a relationship between active smoking and increased risk of COVID-19-associated symptoms. Among the SARS-CoV-2-positive patients, smokers were more likely to need hospitalization than non-smokers [37]. The presence of some comorbidities has been related with COVID-19 severity [38, 39]. Our results showed that the presence of comorbidity in asymptomatic patients is linked to the presence of antibodies, especially in patients with hypertension.

Fever, anosmia, ageusia, and muscle aches were the main symptoms linked to antibody positivity. Anosmia and ageusia had the strongest correlation, while the presence of fever was related to antibody positivity in a Swiss study [35]. The virus-specific IgG, IgM, and IgA titers were not significantly different between the patients with COVID-19 symptoms and those without. This finding was consistent with kinetic studies [40].

The presence of IgA antibodies was indicative of mucosal immunity. In the first weeks after symptom onset, SARS-CoV-2 neutralization is more strongly associated with IgA than IgM or IgG [40]. Older patients have higher IgA levels, which may be due to previous immune memory to pre-infection with other coronaviruses [41]. Despite the lack of statistical significance, the fact that non-smokers had higher IgA levels and two-thirds of those with positive antibodies were non-smokers, the null hypothesis in this relationship cannot be dismissed. A probable explanation is the limited sample size. Further research is required to assess this relationship. While there is no study on the impact of smoking on mucosal IgA or serum IgA, a comparison between smokers with chronic obstructive pulmonary disease (COPD) and healthy smokers found that immune switching to IgG was more common among smokers with COPD. In contrast, switching to IgA was more common among healthy smokers [42]. A study on immune switching in patients with COVID-19 should be conducted.

This study has several novelties. To the best of our knowledge, it is the only study to date that shows a relationship of total antibodies and specific SARS CoV-2 antibodies with both background characteristics and symptom history in asymptomatic patients. Antibody testing was performed a long period after the pandemic onset in low-income countries with an ongoing political conflict and limited COVID-19 management resources, including vaccines. This was performed a long time after it was conducted in developed countries. However, this study had some limitations. First, the survey estimates may have been affected by false-positive (the test result was positive, but the person did not have SARS-CoV-2 antibodies) or false-negative test results (the person had SARS-CoV-2 antibodies, but the test did not detect them). Second, the seroprevalence is representative of only the study period, study site, and testing methods used in the study. Certain sub-analyses did not differ significantly, due to small numbers in some strata, leading to Type II errors.

## Conclusion

The seroprevalence of SARS-CoV-2 antibodies was high among PHC center attendees and was higher among females, non-smokers, those with a COVID-19 contact history, and areas with a high COVID-19 prevalence. However, considering that almost three-quarter of this population remains susceptible, maintaining public health measures and encouraging access to immunization is critical in protecting these people. Specific long-term immune antibodies, primarily IgG and IgA, were detected even after six months of the disease. To characterize the prevalence and trend of COVID-19 over time, continuous monitoring and testing of SARS-CoV-2 antibodies is recommended. Given the timeline of this study, longitudinal studies are needed to determine whether SARS-CoV-2-specific IgA, IgM, and IgG will persist for a longer time in patients with acute COVID-19 than in asymptomatic patients.

## Supporting information

S1 Data Row data file. (XLSX)
